# Prognostic Value of Serum Lactate Dehydrogenase in Renal Cell Carcinoma: A Systematic Review and Meta-Analysis

**DOI:** 10.1371/journal.pone.0166482

**Published:** 2016-11-18

**Authors:** Jie Shen, Zhen Chen, Qianfeng Zhuang, Min Fan, Tao Ding, Hao Lu, Xiaozhou He

**Affiliations:** Department of Urology, The Third Affiliated Hospital of Soochow University, Changzhou, Jiangsu Province, China; National Institute of Health, UNITED STATES

## Abstract

**Background:**

Recently, many studies have shown that the serum lactate dehydrogenase (LDH) level is related to the prognosis of renal cell carcinoma (RCC). We launched this meta-analysis to assess the prognostic value of serum LDH in patients with RCC.

**Methods:**

We searched PubMed, Embase and Web of Science for information on serum LDH and the outcome of RCC through June 14, 2016. The hazard ratio (HR) and its 95% confidence interval (CI) for overall survival (OS) and progression-free survival (PFS) were extracted and integrated from the matching studies.

**Results:**

A total of 29 studies including 6629 patients with RCC were incorporated in this meta-analysis. Patients whose serum LDH levels were elevated had a lower OS (HR = 2.13, 95% CI = 1.69–2.69, *P* < 0.001). Meanwhile, the pooled data showed that a higher serum LDH level was a negative prognostic factor for PFS (HR = 1.74, 95% CI = 1.48–2.04, *P* < 0.001). Furthermore, subgroup analyses indicated elevated serum LDH was associated with poor survival in different tumor types. Elevated serum LDH was significantly associated with worse prognosis for patients with all stages of RCC (OS, HR = 2.41, 95% CI = 1.09–5.33), metastatic RCC (OS, HR = 2.62, 95% CI 1.57–2.59; CSS, HR = 1.79, 95% CI 1.49–2.15), and non-metastatic RCC (OS, HR = 3.67, CI = 1.33–10.13). Besides, elevated serum LDH also indicated a worse prognosis in subgroups of cut-off values, analysis types and ethnicity.

**Conclusions:**

Our results show that serum LDH levels are associated with the outcomes of RCC and can be used as a valuable biomarker for monitoring prognoses.

## Introduction

Renal cell carcinoma (RCC) is a common malignant tumor. The estimated number of new cases of RCC in the United States in 2015 was 61560, representing approximately 3.7% of all new cases, and the estimated deaths were 14080, accounting for nearly 2.4% of the total cancer mortality[1]. Early-stage RCC patients treated with nephrectomy may have a satisfying five-year survival expectancy[2, 3]. Once metastasis occurs, molecular-targeted therapy and immunotherapy are the only ways to treat RCC due to its poor response to radiotherapy and chemotherapy[4–9]. TNM staging is typically used to predict the survival of RCC patients in clinical medicine. Relying on this index, we can propose individualized treatments. However, patients with RCC in the same stage may suffer different outcomes, which urges us to find new and more precise biomarkers to assess the prognosis of RCC.

Aerobic glycolysis is the most prominent characteristic of a cancer cell[10]. A large amount of lactate is produced during this process. Lactate dehydrogenase (LDH) is a glycolysis enzyme that catalyzes the conversion of pyruvate into lactate and that could potentially play an important role in tumor metabolism. In recent years, a large number of studies have demonstrated that serum LDH levels are linked to the prognosis of RCC[11–15]. LDH could be a cheap and simple prognostic index that could be applied in everyday oncologic clinical practice rather than to stratify patients in clinical trials. However, due to different conditions, parameters and other factors included in the studies, the prognostic value of serum LDH in patients with RCC is still inconsistent. Therefore, we conducted this systematic literature review and meta-analysis to research the relationship between serum LDH levels and the prognosis of RCC.

## Materials and Methods

### Search strategy

PubMed, Embase and Web of Science were searched systematically through June 14, 2016. The terms included in the search strategy were as follows: “lactate dehydrogenase or LDH” (all fields), “kidney cancer or renal cancer or renal carcinoma or renal cell carcinoma” (all fields) and “prognosis or prognostic or survival or outcome” (all fields). To guarantee the validity of the retrieved reference lists, each article was screened by two different researchers (Jie Shen and Zhen Chen).

### Selection criteria

Studies were included in our meta-analysis if the following criteria were met: (1) the patients suffered from RCC only, without other malignant tumors; (2) the serum LDH levels were calculated, and the relationship between serum LDH and RCC was mentioned in the article; (3) the values for the hazard ratio (HR) and its 95% confidence interval (CI), overall survival (OS) or disease-free survival (DFS) were mentioned or could be extrapolated; and (4) the studies were written in English. Studies were excluded based on the following criteria: (1) reviews, letters, comments, case reports and conference abstracts; (2) studies with duplicate data; and (3) missing data for further analysis.

### Data extraction

The data were extracted from the articles by two independent authors (Shen and Chen). Details included author, year of publication, country, patient numbers, age of patients, duration of follow-up care, ethnicity, cut-off value, T stage, Furman grade, treatment method, HR and relative 95% CI. In case survival data could only be found in Kaplan–Meier curves, we had to use software designed by Jayne F Tierney and Matthew R Syde[16] to digitize and extract HR values and 95% CIs. Moreover, disputed data were only included after discussion between both authors.

### Statistical analysis

High and low levels of serum LDH were determined according to the cut-off values provided in the articles. In addition, the pooled HRs and their 95% CIs were estimated to evaluate the effective impact of the serum LDH level on survival. We found an observed HR above 1 to be indicative for a poorer prognosis for RCC patients with high serum LDH level. Heterogeneity of combined HRs was tested by applying a Cochran’s Q test and Higgins I-squared statistics. If the *P* value was less than 0.05 and/or *I*^*2*^ was larger than 50%, indicative for a significant heterogeneous distribution, a random-effects model (DerSimonian-Laird method) was used. Otherwise, a fixed-effects model (the Mante-Haenszel method) was implemented. The factors that may have led to the existence of heterogeneity were further analyzed. Publication bias was assessed by using Begg’s and Egger’s tests. All data analyses were performed with STATA 12.0 (Stata Corporation, College Station, TX, USA). Applying a two-sided test, a *P* value lower than 0.05 was defined as statistically significant.

## Results

### Search results

**Fig 1:**
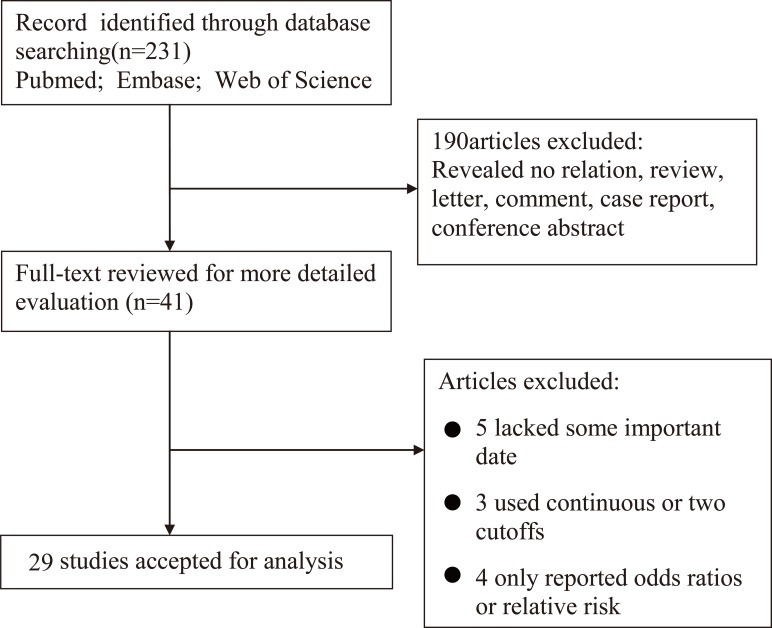
Flow diagram of the study selection process.

The process of searching and filtering of articles is shown in Fig 1. Initially, a total of 231 articles were listed. After scanning the titles, abstracts, publication categories and full text of each article, 41 articles were shortlisted. Among these, 12 articles were excluded (five lacked some important data, three used continuous or two cut-offs, and four only reported odds ratios or relative risk). Finally, 29 articles, representing 6629 patients with RCC, were included in this meta-analysis[11–15, 17–40].

### Study characteristics

The characteristics of the 29 eligible studies are presented in Table 1. The studies were originally published between 2002 and 2016. The 6629 patients were from Canada, China, Czech, Denmark, Germany, Italy, Japan, Netherlands, Poland, Turkey and the USA. Furthermore, Asian nationals account for 19.4% of the patients (1289 patients) and Caucasian patients account for the remaining 80.6% (5340 patients). In this study, 2287 patients in 12 articles were treated with multiple therapies, while 3465 patients in another 12 articles were treated with targeted therapy only. The remaining 1123 patients received surgery only. Meanwhile, OS was mentioned in 25 articles, and PFS was found in 9 articles.

**Table 1:** Main characteristics of all studies included in the meta-analysis.

Author	Year	Country	Number	Age	Follow-up	Ethnicity	Cut-off value	T stage	Furman	Tumor type	Survival analysis	Source of HR	Multivariate analysis	Treatment
Chrom	2016	Poland	266	61(22–85)	46.1(41.2–51)	Caucasian	ULN	45/68/109/8	13/127/71/28	mRCC	OS	Report	yes	targeted therapy
Song	2016	China	74	51.4(20.1–86.8)	38.3(2.3–53.9)	Asian	1.5ULN	27/35/11/1	NR	mRCC	OS	Report	yes	MT
Song	2016	China	155	55.3(17.9–86.8)	36.3(4.3–110.5)	Asian	1.5ULN	48/36/11	NR	mRCC	OS/PFS	Report	yes	targeted therapy
Matrana	2015	USA	88	65(34–90)	29.3(25.4–33.2)	Caucasian	1.5ULN	19/42	NR	mRCC	PFS	SC	yes	surgery
Fukushima	2015	Japan	92	65(37–91)	19(1–142)	Asian	1.5ULN	NR	NR	mRCC	OS	Report	yes	MT
Sasaki	2015	Japan	126	67	30.8	Asian	250U/L	NR	NR	All stage	OS	Report	yes	MT
Bodnar	2015	Poland	58	60(41–78)	NR	Caucasian	ULN	NR	NR	mRCC	OS	Report	yes	targeted therapy
Kubackova	2014	Czech	836	59(21–83)	NR	Caucasian	1.5ULN	NR	NR	All stage	OS/PFS	Report	no	targeted therapy
Girgis	2014	Canada	385	NR	NR	Caucasian	NR	190/35/49/33/78(x)	31/180/103/22/49(x)	Non-mRCC	OS	SC	yes	surgery
Malik	2014	USA	70	56.5(44–76)	NR	Caucasian	1.5ULN	NR	NR	All stage	OS	Report	yes	MT
Poprach	2014	Czech	319	62(45–77)	15	Caucasian	1.5ULN	NR	NR	mRCC	OS/PFS	Report	no	MT
Amato	2014	USA	57	NR	NR	Caucasian	1.5ULN	NR	13/39(1,2–3,4)	All stage	OS	Report	yes	MT
Cetin	2014	Turkey	59	60 (34–80)	15(2–59)	Caucasian	ULN	NR	NR	mRCC	OS	Report	no	MT
Atkinson	2014	USA	185	Over 18	NR	Caucasian	927	NR	NR	mRCC	OS/PFS	Report	yes	targeted therapy
Nakano	2013	Japan	36	65.7	13(2–48)	Caucasian	200U/L	NR	NR	mRCC	PFS	Report	yes	targeted therapy
Kamba	2013	Japan	144	62.9(19–86)	2–218	Caucasian	1.5ULN	NR	NR	mRCC	PFS	Report	yes	MT
Motzer	2013	USA	1059	60(24–87)	NR	Caucasian	1.5ULN	NR	NR	mRCC	OS/PFS	Report	yes	targeted therapy
Armstrong	2012	USA	404	59.4(23–86)	NR	Caucasian	ULN	NR	NR	mRCC	OS	Report	yes	targeted therapy
Du	2012	China	286	55.72(28–77)	NR	Asian	1.5ULN	165/55/52/4	17/134/112/23	mRCC	OS	Report	yes	surgery
Shinohara	2011	Japan	473	64(32–87)	18	Asian	1.5ULN	NR	NR	mRCC	OS	Report	yes	MT
Abel	2011	USA	75	60(23–80)	15	Caucasian	ULN	14/16/34/11	12/20/4/32/7(Ⅱ/Ⅲ/Ⅳ/unknown/high grade)	mRCC	OS	Report	yes	targeted therapy
Zhang	2011	China	83	51(27–75)	27(12–46)	Asian	315IU/L	NR	NR	mRCC	PFS	Report	yes	targeted therapy
Aben	2011	Netherlands	328	67.6±11	NR	Caucasian	1.5ULN	NR	14/32/43/13/226 (uknown)	mRCC	OS	Report	yes	MT
Richey	2011	USA	188	60.8(18.2–83.9)	13.1(1.0–64.4)	Caucasian	ULN	45/28/89/26	NR	mRCC	OS	Report	yes	targeted therapy
Jeppesen	2010	Denmark	120	58.3(29–73)	48–72	Caucasian	1.5ULN	NR	NR	mRCC	OS	Report	yes	MT
Donskov	2006	Denmark	120	57(19–74)	57(32–73)	Caucasian	1.5ULN	NR	NR	mRCC	OS	Report	yes	targeted therapy
Peccatori	2005	Italy	70	NR	10	Caucasian	300U/L	NR	NR	All stage	OS	Report	yes	surgery
Lehmann	2004	Germany	48	63(35–82)	125.3(33.4–156)	Caucasian	183U/L	44761	NR	Non-mRCC	OS	Report	no	surgery
Atzpodien	2002	Germany	425	NR	20(0–157)	Caucasian	220U/L	NR	NR	mRCC	OS	Report	yes	MT

Abbreviation: RCC renal cell carcinoma, mRCC metastatic renal cell carcinoma, Non-mRCC non- metastatic renal cell carcinoma, OS overall survival, PFS progression-free survival, HR hazard ratio, NR not report, MT multiple therapy, SC survival curve, ULN upper limits of normal, x status unknown.

### Quality assessment

We assessed the quality of the 29 eligible studies included in our meta-analysis following the guideline of the Newcastle-Ottawa Scale (NOS)[41]. The quality of the studies varied from a score of 5 to 9, with a mean of 6. A higher score suggested the use of better methodologies. Therefore, all 29 studies were included in the subsequent analysis.

### Overall survival

**Fig 2:**
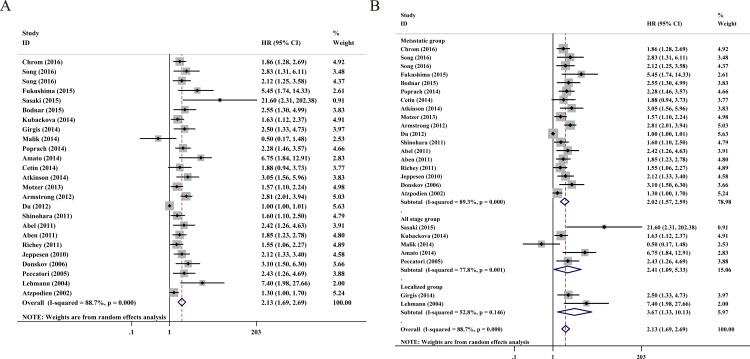
**A.** Forest plots of studies evaluating hazard ratios of elevated serum LDH level in all renal cell carcinoma (RCC) for overall survival. **B.** Forest plot of the relationship between elevated serum LDH level and overall survival in patients with different tumor types.

In total, 25 studies, including 6278 RCC patients, provided valid data for OS and are presented in Fig 2A. We used a random model to pool the HRs because of statistical heterogeneity (*I*^*2*^ = 88.7%, *P* < 0.001). The pooled data demonstrated that high serum LDH predicts worse OS (HR = 2.13, 95% CI = 1.69–2.69, *P* < 0.001).

In order to further test for heterogeneity among the studies, we performed this meta-analysis on discrete subgroups, including ethnicity, cut-off value, analysis type and tumor type. High serum LDH was linked to a worse OS for all stages of RCC (HR = 2.41, 95% CI = 1.09–5.33, *P* = 0.03), metastatic RCC (HR = 2.02, 95% CI = 1.57–2.59, *P* <0.001) and non-metastatic RCC (HR = 3.67, 95% CI = 1.33–10.13, *P* = 0.012), as shown in Table 2 and Fig 2B. Serum LDH also showed a statistically significant connection with poor OS in the remaining subgroups: Asian RCC patients (HR = 2.22, 95% CI = 1.27–3.87, *P* = 0.005), Caucasian RCC patients (HR = 2.06, 95% CI = 1.73–2.44, *P* <0.001), multivariate analysis (HR = 2.11, 95% CI = 1.63–2.71, *P* <0.001), univariate analysis (HR = 1.98, 95% CI = 1.52–2.56, *P* <0.001), 1.5 ULN (HR = 1.96, 95% CI = 1.43–2.68, *P* <0.001), and others (HR = 2.21, 95%CI = 1.73–2.83, *P* <0.001).

**Table 2:** Pooled hazard ratios for OS according to subgroup analyses.

Outcome subgroup	No. of patients	No. of studies	HR (95% CI)	*P* value	Model	heterogeneity	
						*I*^*2*^ (%)	*P*
**Overall survival**	6278	25	2.13(1.69–2.69)	<0.001	random	88.7%	<0.001
**Ethnicity**							
Asian	1206	6	2.22(1.27–3.87)	0.005	random	86.4%	<0.001
Caucasian	5072	19	2.06(1.73–2.44)	<0.001	random	54.3%	0.003
**Cut-off value**							
1.5ULN	4414	13	1.96(1.43–2.68)	<0.001	random	87.9%	<0.001
Others	1864	12	2.21(1.73–2.83)	<0.001	random	59.4%	0.004
**Analysis type**							
Multivariate	5016	21	2.11(1.63–2.71)	<0.001	random	89%	<0.001
Univariate	1262	4	1.98(1.52–2.56)	<0.001	fixed	43.3%	0.152
**Tumor type**							
All stage	1159	5	2.41(1.09–5.33)	0.03	random	77.8%	0.001
Metastasis	4686	18	2.02(1.57–2.59)	<0.001	random	89.3%	<0.001
Non-metastasis	433	2	3.67(1.33–10.13)	0.012	random	52.8%	0.146

Abbreviation: OS = overall survival; HR = hazard ratio; CI = confidence interval.

### Sensitivity analysis

Each study was tested for the impact of a single study on the pooled HRs. One study deviated from the rest and may have influenced the stability of the result. After excluding this study, the pooled HRs and 95% CI for OS were 2.11 and 1.8–2.48, which is a further indication of the strength of our meta-analysis.

### Progression-free survival

**Fig 3:**
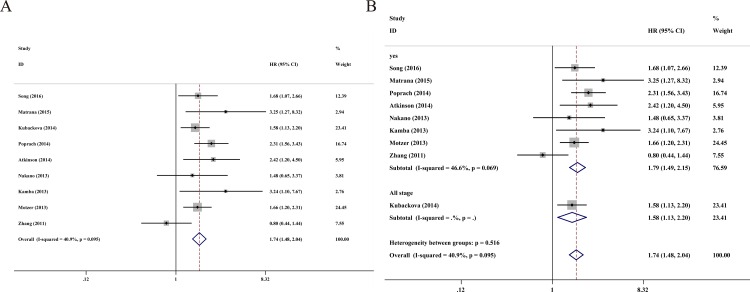
**A.** Forest plots of studies evaluating hazard ratios of elevated serum LDH level in all renal cell carcinoma (RCC) for progression-free survival. **B.** Forest plot of the relationship between elevated serum LDH level and progression-free survival in patients with different tumor types.

Next, 9 studies contained a total 2905 RCC patients with valid data for PFS, as showed in Fig 3A. A fixed-effect model was used in the analysis of PFS due to the absence of statistical heterogeneity (*I*^*2*^ = 40.9%, *P* = 0.095). Additionally, the pooled data demonstrated that high serum LDH predicts worse PFS (HR = 1.74, 95% CI = 1.48–2.04, *P* < 0.001). A subgroup analysis was conducted simultaneously in order to evaluate the heterogeneity of these studies (Table 3 and Fig 3B). A separate meta-analysis, including 5 studies and 2487 Caucasian patients, had a pooled HR for PFS of 1.87 (95% CI = 1.55–2.25; *P* < 0.001) with moderate heterogeneity among studies (*I*^*2*^ = 11%, *P* = 0.343). Other subgroups, such as the analysis type and tumor type for PFS, were shown to be statistically linked to serum LDH.

**Table 3:** Pooled hazard ratios for PFS according to subgroup analyses.

Outcome subgroup	No. of patients	No. of studies	HR (95% CI)	*P* value	Model	heterogeneity	
						*I*^*2*^ (%)	*P*
**progression-free survival**	2905	9	1.74(1.48–2.04)	<0.001	fixed	40.9%	0.095
**Ethnicity**							
Asian	418	4	1.48(0.89–2.48)	0.133	random	57.2%	0.072
Caucasian	2487	5	1.87(1.55–2.25)	<0.001	fixed	11%	0.343
**Cut-off value**							
1.5ULN	2601	6	1.84(1.54–2.19)	<0.001	fixed	5.7%	0.38
Others	304	3	1.4(0.7–2.79)	0.344	random	67.3%	0.047
**Analysis type**							
Multivariate	1750	7	1.67(1.36–2.06)	<0.001	fixed	45.8%	0.086
Univariate	1155	2	1.88(1.30–2.73)	0.001	random	52%	0.149
**Tumor type**							
All stage	836	1	1.58(1.13–2.20)	0.007	fixed	-	-
Metastasis	2069	8	2.02(1.57–2.59)	<0.001	fixed	46.6%	0.069

Abbreviation: DFS = disease-free survival; HR = hazard ratio; CI = confidence interval.

### Publication bias

**Fig 4:**
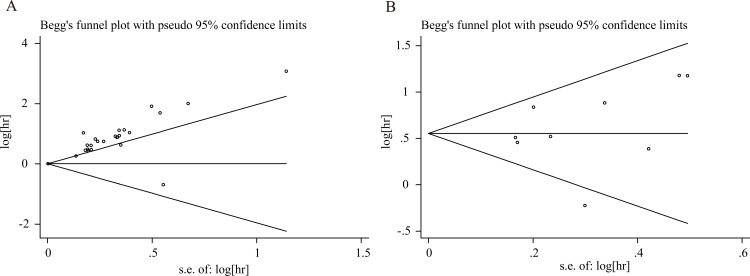
Funnel plots for the evaluation of potential publication bias. (A) Overall survival for all renal cell carcinoma; (B) Progression-free survival for all renal cell carcinoma.

We assessed the publication bias of OS and PFS. The publication bias of all studies was evaluated using funnel plots and Egger’s and Begg’s tests. Funnel plots of OS and PFS are presented in Fig 4A and 4B, respectively. The figures show an existing publication bias for OS (*P* <0.01 using Egger’s test), while a publication bias for PFS does not exist (*P* = 0.531 using Egger’s test).

## Discussion

Aerobic oxidation and glycolysis are two different pathways of glucose metabolism responding to distinct demands of oxygen. In most mammalian cells, glycolysis is inhibited under aerobic conditions and mitochondria oxidize pyruvate producing mainly CO_2_ and H_2_O. This phenomenon is known as the Pasteur effect[42]. However, in tumor cells, glycolysis is extremely elevated regardless of oxygen availability, which is called the Warburg effect[10]. As a result of high glycolysis, the production of lactate is enhanced, creating an acidic microenvironment in the tissues. Normal cells become necrotic due to the change of the transmembrane H^+^ gradient and the degradation of the extracellular matrix in an acidic microenvironment[43]. However, tumor cells can resist acidosis and survive in low pH conditions. Meanwhile, acidosis can inhibit the immune response to tumor antigens, which promotes the proliferation and invasion of tumor cells[44].

LDH is an essential substance in the Warburg effect and is available in almost all tissues. It has six different iso-enzymes[45]. These iso-enzymes are assembled in a homo- or hetero-tetramer structure by two protein subunits: LDHA and LDHB. Different combinations of LDHA and LDHB characterize five subtypes of LDH (LDH-1 to LDH-5)[45]. A third protein subunit, which is named LDHC, forms a testis-specific subtype, known as LDH-6[46]. The predominant function of LDH is the catalyzation of the reversible reaction of pyruvate to lactate. NAD^+^ is generated concomitantly alongside this process and is essential for the continuous generation of ATP to keep glycolysis running. Among LDH subtypes, the catalytic efficiency of LDH-5 is the highest[47]. When RCC tissue necrosis occurred, high level of intracellular LDH was released into blood, improving the serum LDH concentration[10]. Moreover, when distant metastasis occurred, tumor cells can disrupt neighboring organs such as liver, lung, and bones. The injury of those organs can also elevate serum LDH level[47–50]. Recently, several studies have proved that reduced LDH expression can inhibit the invasion and metastasis of cancer cells by decreasing their ability to proliferate and their resistance to chemotherapy[51]. In conclusion, drugs targeting LDH may represent new approaches for the treatment of RCC.

To our knowledge, this meta-analysis is the most comprehensive research to synthetically analyze the prognostic value of serum LDH in patients with RCC. We conclude that an elevated serum LDH is an objective biomarker for worse OS and PFS in RCC patients. Meanwhile, subgroup analyses demonstrated that elevated serum LDH remained a good biomarker regardless of ethnic background, analysis type and HR diagnostic method.

However, this meta-analysis has some limitations. First, we only considered articles mentioning the HR and the 95% CI, while other articles were excluded because they only reported odds ratios and relative risk for survival. Second, due to different standards for the use of cut-off values in the articles, the validity of serum LDH as an important role to predict the prognosis of RCC patients may have been affected. Moreover, serum LDH could have been influenced by other non-neoplastic conditions, such as muscular dystrophy, anemia, heart failure, hepatitis and lung disease. Most articles contained in this meta-analysis did not investigate those factors. Regardless, serum LDH is an important and meaningful biomarker to assess the prognosis of patients with RCC.

In conclusion, this meta-analysis demonstrated that an elevated serum LDH level was connected to a poor prognosis for patients with RCC. The serum LDH was a convenient and cost-effective prognostic indicator, which could be utilized to divide risk stratification and formulate individualized treatments for patients with RCC. Considering the limitation of the present analysis, the more detailed mechanism why or how LDH activity in RCC tumors could elevate serum LDH level is still unclear. We will need to conduct further multicenter studies to confirm our findings and explore the mechanism deeply.

## Supporting Information

S1 File PRISMA 2009 Checklist. (DOC)
