# Expression of Concern: Prognostic Value of Serum Lactate Dehydrogenase in Renal Cell Carcinoma: A Systematic Review and Meta-Analysis

**DOI:** 10.1371/journal.pone.0285005

**Published:** 2023-04-21

**Authors:** The PLOS ONE Editors

After this article was published, similarities were noted between this article [1] and submissions by other research groups which call into question the validity and provenance of the reported results.

In response to queries about these concerns, the corresponding author provided the underlying data in S1–S2 Files. They also provided output data (S3 File) from Engauge Digitizer software, developed by Mark Mitchel et al [7], which re-calculated the survival data through Kaplan-Meier curves. The corresponding author stated that for Girgis’ article [6], this (HR = 2.3, 95% CI = 1.24–4.3) is different to the data given in article [1] for Girgis’ article [6] where HR = 2.5, 95% CI = 1.33–4.73. The corresponding author stated that the difference in the two results is due to a deviation in the intercepted points process when drawing Kaplan-Meier curves by hand.

During editorial follow-up, the following errors in article [1] were noted:

- There are two instances in Fig 2 where the I-squared p value is reported as 0.000. This represents cases where p < 0.001, the result of outputs by software StataSE.
- In Table 1:
  - The Nakano study [3] involved Japanese participants; the Ethnicity entry should be Asian.
  - The Lehmann study [4] T stage entry should be 22/7/19.
  - The Matrana study [5] T stage entry should be 19/42(<T4/T4).
- The univariate analyses (HR = 1.63, 95% CI = 1.12–2.37) instead of the multivariate analyses (HR = 2.06, 95% CI = 1.29–3.28) from Kubackova’s research [2] was used. Updated Fig 2A and 2B and an updated Table 2 are provided here where the multivariate analyses in Kubackova’s research [2] is used. The text referring to Table 2 has been updated as follows: ‘High serum LDH was linked to a worse OS for all stages of RCC (HR = 2.57, 95% CI = 1.16–5.67, P = 0.03), metastatic RCC (HR = 2.02, 95% CI = 1.57–2.59, P <0.001) and non-metastatic RCC (HR = 3.67, 95% CI = 1.33–10.13, P = 0.012), as shown in Table 2 and Fig 2B. Serum LDH also showed a statistically significant connection with poor OS in the remaining subgroups: Asian RCC patients (HR = 2.22, 95% CI = 1.27–3.87, P = 0.005), Caucasian RCC patients (HR = 2.09, 95% CI = 1.76–2.49, P <0.001), multivariate analysis (HR = 2.11, 95% CI = 1.63–2.71, P <0.001), univariate analysis (HR = 2.24, 95% CI = 1.69–2.98, P <0.001), 1.5 ULN (HR = 2.01, 95% CI = 1.45–2.78, P <0.001), and others (HR = 2.21, 95%CI = 1.73–2.83, P <0.001).’

**Fig 2:**
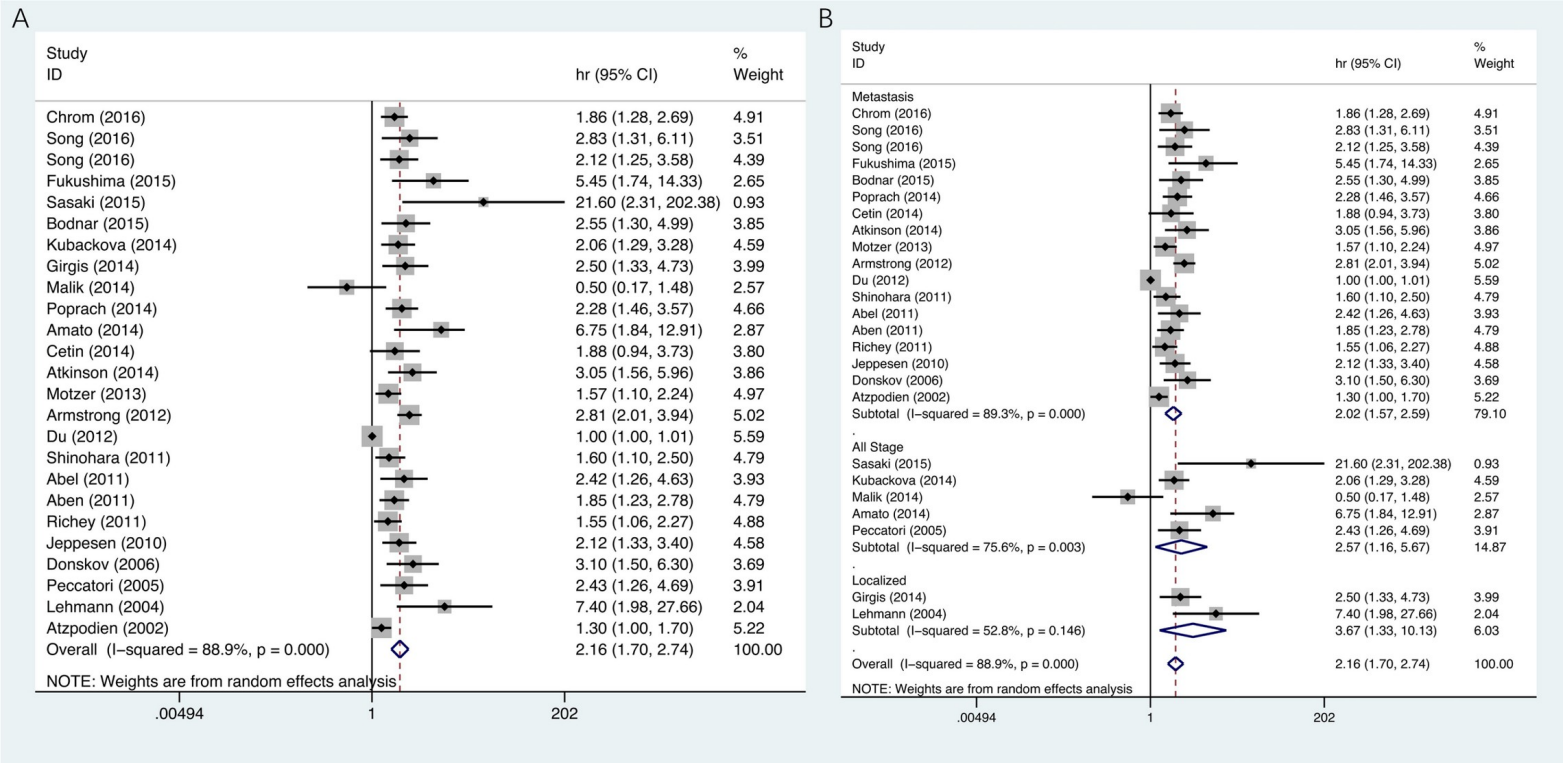
**A.** Forest plots of studies evaluating hazard ratios of elevated serum LDH level in all renal cell carcinoma (RCC) for overall survival. **B.** Forest plot of the relationship between elevated serum LDH level and overall survival in patients with different tumor types.

An expert statistical reviewer assessed the updated Fig 2 compared to the original results and stated that the main conclusions are not affected as the All stage group Subtotal HR (95% CI) in Fig 2B, and the Overall HR (95% CI) in Fig 2A and 2B value differences between using univariate and multivariate from Kubackova’s research [2] are small. The statistical reviewer also noted that many of the results from the other references used are in agreement in the Forest plots in Fig 2A and 2B.

The concerns about specific results were resolved in discussions with the authors and reviewer, and the dataset received from the authors provides a level of assurance as to the provenance of this work. Nevertheless, PLOS remains concerned about similarities between [1] and other publications and so, the *PLOS ONE* Editors issue this Expression of Concern.

**Table 2:** Pooled hazard ratios for OS according to subgroup analyses.

Outcome subgroup	No. of patients	No. of studies	HR (95% CI)	P value	Model	heterogeneity	
						I^2^ (%)	P
**Overall survival**	6278	25	2.16(1.70–2.74)	<0.001	random	88.9%	<0.001
**Ethnicity**							
Asian	1206	6	2.22(1.27–3.87)	0.005	random	86.4%	<0.001
Caucasian	5072	19	2.09(1.76–2.49)	<0.001	random	53.5%	0.003
**Cut-off value**							
1.5ULN	4414	13	2.01(1.45–2.78)	<0.001	random	88.9%	<0.001
Others	1864	12	2.21(1.73–2.83)	<0.001	random	59.4%	0.004
**Analysis type**							
Multivariate	5016	21	2.11(1.63–2.71)	<0.001	random	89%	<0.001
Univariate	1262	4	2.24(1.69–2.98)	<0.001	fixed	15.3%	0.315
**Tumor type**							
All stage	1159	5	2.57(1.16–5.67)	0.03	random	75.6%	0.003
Metastasis	4686	18	2.02(1.57–2.59)	<0.001	random	89.3%	<0.001
Non-metastasis	433	2	3.67(1.33–10.13)	0.012	random	52.8%	0.146

## Supporting information

S1 File The underlying data used for the analysis in this article [1]. (XLSX)

S2 File The underlying data used for the analysis in Fig 2A this article [1]. (XLSX)

S3 File Output data from Engauge Digitizer software. (CSV)
